# Electroconvulsive Therapy for Traumatic Brain Injury and Schizoaffective Disorder

**DOI:** 10.7759/cureus.16390

**Published:** 2021-07-14

**Authors:** Ryan Adachi, Chong Yang

**Affiliations:** 1 College of Osteopathic Medicine, Touro University California, Vallejo, USA; 2 Psychiatry, California Department of State Hospitals, Napa, USA

**Keywords:** electroconvulsive therapy, ect, traumatic brain injury, tbi, psychosis, schizoaffective disorder

## Abstract

Traumatic brain injury (TBI) is a source of disability and mortality with high rates of psychiatric disorders. Patients with comorbid TBI and psychiatric disorders may be safely treated with electroconvulsive therapy (ECT). In this case report, we present a 34-year-old man with the diagnosis of major neurocognitive disorder due to traumatic brain injury and schizoaffective disorder, bipolar type. He received an index course of 19 ECT treatments with clinical improvements in his psychosis, mood, and cognition. This case may support the utilization of ECT for patients with comorbid TBI and schizoaffective disorder.

## Introduction

Traumatic brain injury (TBI) is defined as an alteration in brain function, or other evidence of brain pathology, caused by an external force [1]. It typically presents with a loss of (or decreased) consciousness, memory impairment, neurologic deficits, or changes in mental status. Alternatively, TBI may manifest with delayed or the absence of clinical symptoms despite evidence of brain pathology as determined by imaging or laboratory investigations [1]. Regardless of the clinical presentation, TBI is a significant source of disability and mortality. The Global Burden of Disease reported that the global annual incidence and prevalence of TBI was estimated at 27.08 million and 55.5 million, respectively [2]. A cohort study revealed that patients with severe head injury are at an increased risk of mortality for at least 13 years after the trauma [3].

In addition to the cognitive impairments, patients with TBI experience high rates of psychiatric disorders, including depression, anxiety, mania, irritability, aggression, panic or obsessive-compulsive tendencies, and drug abuse [4,5]. Therefore, psychiatric treatment includes the utilization of psychotropic medication, therapy, and electroconvulsive therapy (ECT). ECT is a procedure, performed under general anesthesia, that alleviates the symptoms of mood disorders, psychosis, and other psychiatric disorders. It is a safe procedure with a mortality rate of two to four deaths per 100,000 treatments [6,7]. This death rate is smaller compared with the spontaneous death rate in the general population [7].

The primary concern with ECT is the potential worsening of cognition. The potential cognitive effects of ECT include transient confusion, anterograde amnesia, and retrograde amnesia [8]. Despite this limitation, Krystal and Coffey reported that ECT can be safely administered to patients with a history of brain injury [9]. Similarly, a case series of 11 patients with closed head injuries and various psychiatric disorders supported the use of ECT [10]. These patients received ECT treatments and demonstrated resolution of their neuropsychiatric symptoms. Finally, Srienc et al. reviewed three case reports of patients with TBI and post-TBI depression. These patients also received ECT treatments with good outcomes [11].

Unfortunately, the literature on ECT treatments for TBI and concurrent psychosis is limited. Johnson and Ward reported the alleviation of psychotic symptoms in a patient with TBI and psychosis with ECT treatments [12]. Otherwise, to our knowledge, there are no available reports of ECT treatment for patients diagnosed with TBI and schizoaffective disorder. In the following case report, we present a patient diagnosed with TBI and schizoaffective disorder in the context of ECT treatment.

## Case presentation

A 34-year-old man was hospitalized in a state hospital with the diagnosis of major neurocognitive disorder due to traumatic brain injury and schizoaffective disorder, bipolar type. His parent provided collateral information which includes a significant history of repeated head trauma. The parent shared that the patient received his first head injury while playing football in his teenage years. His parent noted approximately 10 additional incidents of head trauma and added that he developed significant cognitive impairment over the years. He experienced difficulties with attention, concentration, and memory. In the hospital, the staff observed his cognitive difficulties, and the patient was administered psychological testing, including the Wechsler Adult Intelligence Scale, Fourth Edition (WAIS-IV). The collateral information, clinical observation, and results of the psychological testing supported the diagnosis of major neurocognitive disorder due to traumatic brain injury. 

The patient’s diagnosis of schizoaffective disorder, bipolar type, was supported by the presence of auditory hallucinations, disorganized thinking, delusions, irritability, and impulsivity. Furthermore, the patient engaged in more than 30 behavioral incidents of psychotic agitation over the course of a year. Behavioral incidents include assaults towards staff/patients and the swallowing of batteries. These dangerous behaviors have led to his frequent placement in five-point restraints and transfers out of the hospital for additional medical workup and treatment. The patient's symptoms of TBI predated his symptoms of psychosis. His cognitive symptoms developed during his teenage years, whereas his symptoms of schizoaffective disorder developed in his early 20s. 

The patient did not respond to numerous psychotropic medications. He continued to experience psychotic symptoms despite adherence to olanzapine (up to 30 mg/day), quetiapine (up to 900 mg/day), haloperidol decanoate (up to 300 mg every month), risperidone (up to 4 mg/day), aripiprazole (up to 30 mg/day), chlorpromazine (up to 300 mg/day), loxapine (up to 200 mg/day), fluphenazine decanoate (up to 100 mg every two weeks), and clozapine (up to 900 mg/day). Similarly, he continued to experience mood symptoms despite adherence to divalproex sodium (up to 4000 mg/day), topiramate (up to 400 mg/day), lithium (up to 1200 mg/day), and valproic acid (up to 4500 mg/day). Furthermore, he experienced side effects with higher doses of clozapine (sialorrhea) and fluphenazine (tremors). He was referred for ECT treatment while remaining adherent to clozapine 350 mg at night and fluphenazine decanoate 100 mg every two weeks. 

The patient was treated on the Thymatron System IV (Venice, FL: Somatics, LLC) with a pulse width of 0.5 milliseconds and his energy setting was based on titration. Other treatment parameters include bilateral stimulus electrode placement, 1 milligram/kilogram (mg/kg) of methohexital as the general anesthetic, and 1 mg/kg of succinylcholine as the neuromuscular blockade. The seizure threshold was established at 10% energy (or 50 mC) at the first ECT treatment session. All subsequent treatments were delivered at 2x the seizure threshold (20% energy or 100 mC). The patient received 19 therapeutic ECT treatments with this treatment protocol. We recorded a motor seizure (confirmed by electroencephalogram monitoring) of greater than 20 seconds for all ECT treatments. The results of each treatment are summarized in Table 1.

**Table 1:** Parameters at each treatment session

Treatment	Energy (%)	Charge (mC)	Motor seizure (sec)	EEG seizure (sec)
1; first attempt	5	25	N/A	N/A
1; second attempt	10	50	80	104
2	20	100	50	96
3	20	100	53	92
4	20	100	65	93
5	20	100	59	88
6	20	100	45	86
7	20	100	39	72
8	20	100	42	63
9	20	100	45	90
10	20	100	38	54
11	20	100	43	63
12	20	100	32	61
13	20	100	37	59
14	20	100	29	49
15	20	100	46	85
16	20	100	37	52
17	20	100	29	45
18	20	100	41	80
19	20	100	32	46

The patient responded well to his index course of ECT treatments. He reported significant relief from his psychotic and mood symptoms. He did not report auditory hallucinations or express delusional content. He presented with organized thinking and a stable mood. Finally, the patient did not engage in additional behavioral incidents of psychotic agitation. There were no incidents of assaults towards staff/patients or self-harmful acts such as the swallowing of batteries during his index course of ECT treatments. He remained on clozapine 350 mg at night and fluphenazine decanoate 100 mg every two weeks during and after the course of ECT treatments.

## Discussion

Prior to starting his ECT treatments, there were initial concerns about the potential worsening of cognition in our patient with TBI and pre-existing cognitive impairment. Koola reported that any ECT-induced cognitive impairments, such as retrograde amnesia and anterograde amnesia, may be treated with a combination of galantamine and memantine [13]. Fortunately, our patient presented with improvements in the organization of his thinking and did not require such intervention. We attempted to confirm his cognitive improvements with objective measures. However, he did not cooperate with any formal cognitive testing such as the Mini-Mental State Exam (MMSE) or Montreal Cognitive Assessment. At the patient’s request and our clinical observation of his improved cognition, we elected to resume his ECT treatments without any formal cognitive assessments. He recalled the names of all members of the ECT team (ECT psychiatrist, nurse, and anesthesiologist). He also recalled the contents of our previous conversations. Our findings of no worsening of cognition appear to be consistent with the findings in the literature. As per the literature review, Kant et al. noted no significant decline in post-ECT Neurobehavioral Cognitive Status Examination and MMSE scores for patients with head injuries receiving ECT treatment [10]. Similarly, Sanghani et al. concluded that psychotic patients experience mild and transient cognitive effects, whereas others demonstrated improvement in cognition with ECT [14].

In the consideration of risks and benefits, our case report supports the utilization of ECT treatments for patients with TBI and schizoaffective disorder. Our patient obtained clinical benefits without experiencing any cognitive impairments of ECT. However, additional studies including the inclusion of larger sample size and objective measures of cognition are necessary to confirm our findings.

## Conclusions

Patients with a TBI may experience various psychiatric symptoms including cognitive impairment. These patients may benefit from ECT. Unfortunately, the literature is limited on the efficacy of ECT for patients diagnosed with comorbid TBI and psychosis. We presented a patient with the diagnosis of major neurocognitive disorder due to traumatic brain injury and schizoaffective disorder, bipolar type. The patient obtained clinical benefits without a decline in cognition. Our case report supports the utilization of ECT for patients with TBI and psychosis. However, additional studies are necessary to support our findings.
